# Acute intestinal necrosis due to multiple thrombosis in COVID-19 patient. Report of a case

**DOI:** 10.1186/s40792-022-01495-6

**Published:** 2022-07-19

**Authors:** Hirotsugu Morioka, Michitoshi Goto, Haruka Tanaka, Hirotaka Momose, Kazuyoshi Fujino, Toshiaki Hagiwara, Jun Aoki, Michihiro Orihata, Kotaro Kaneko

**Affiliations:** 1 Department of Surgery, Tokyo Rinkai Hospital, 1-4-2 Rinkai-Cho, Edogawa-Ku, Tokyo, 134-0086 Japan; 2 Department of Pediatric Surgery, Juntendo University, Tokyo, Japan

**Keywords:** COVID-19, Thrombosis, Intestinal ischemia, Intestinal necrosis

## Abstract

**Background:**

While thrombosis is a well-known complication of coronavirus disease 2019 (COVID-19) infection, reports on intestinal necrosis due to intestinal ischemia caused by thrombosis are extremely rare. We herein report a case of intestinal necrosis due to multiple thrombosis in a COVID-19 patient.

**Case presentation:**

The patient was a 64-year-old man. He was admitted to hospital after being diagnosed with COVID-19, the severity was classified as moderate II. Nasal High Flow™ management was conducted along with treatment with tocilizumab, remdesivir, and dexamethasone. Heparin was also administered due to high D-dimer values. As abdominal pain appeared from the 6th day of hospitalization, contrast-enhanced CT was performed, which confirmed multiple thrombosis in the aorta. However, no obvious intestinal ischemia was found. On the 10th day of hospitalization, the patient’s abdominal pain was exacerbated. Upon re-evaluation by CT, he was diagnosed with perforative peritonitis due to ileal ischemic necrosis and emergency surgery was performed. Intraoperative examination revealed perforation due to necrosis at multiple sites in the ileum; thus, partial ileectomy was carried out. Pathological findings also revealed discontinuous multiple intestinal necrosis due to the frequent occurrence of thrombosis. Following surgery, the patient recuperated and was discharged after ventilator management and multimodal therapy at the ICU.

**Conclusions:**

Thrombosis due to COVID-19 complications is rare in the intestinal tract, but also occur. Its initial symptoms might not be captured by CT images, therefore caution is required.

## Case presentation

The World Health Organization has declared novel coronavirus disease 2019 (COVID-19) a global public health emergency. Although respiratory symptoms predominate in COVID-19, thrombosis can also occur. Acute mesenteric ischemia is a less common but devastating complication of COVID-19 disease. We herein report a case of acute intestinal necrosis due to multiple thrombosis in a COVID-19 patient.

### Family history: nothing of note

History of present illness: A 64-year-old man with a history of hypertension (Taking calcium channel blocker) presented to the emergency room with a 1-week history of a persistent fever, shortness of breath, and cough.

He was admitted to the hospital with a diagnosis of COVID-19 (Moderate II [1]) because he had pneumonia on computed tomography (CT) findings, respiratory failure with SpO2 ≤ 93% and needed oxygen.

On admission, he was 172.5 cm tall, weighed 56.6 kg, and had a body mass index of 19.0. His blood pressure was 154/74 mmHg, heart rate was 98 beats per minute, and body temperature was 37.3 °C. SpO_2_ was 89% and PaO_2_ was 58 Torr in room air. Chest findings were crepitant rale in both lungs, and the abdomen was flat and soft with no tenderness. The lower extremities showed no edema, and Homans sign was negative. Blood tests revealed WBC 6120/μl, CRP 7.21 mg/dl, and D-dimer 6.5 μg/ml.

## Clinical course

**Fig. 1:**
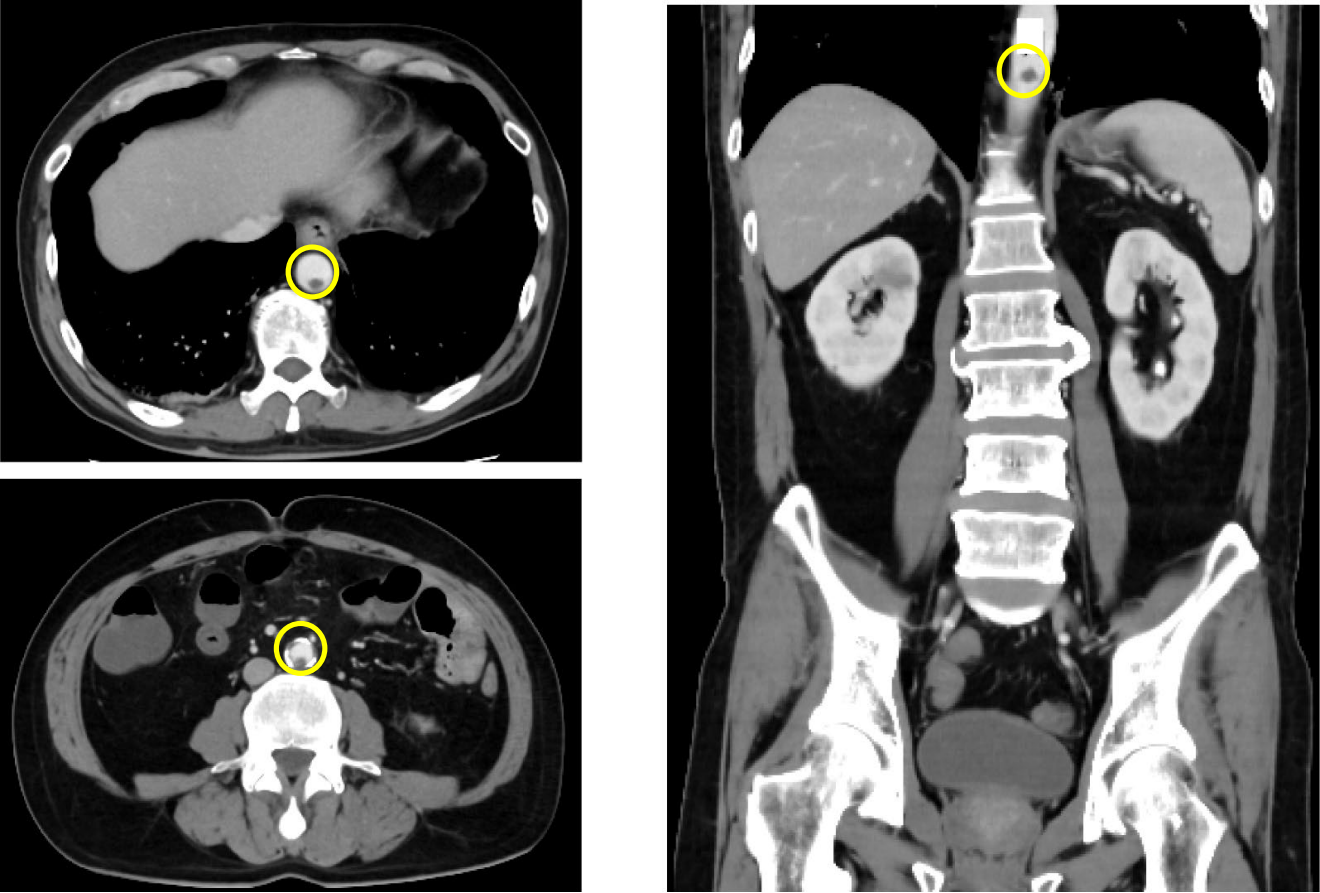
Abdominal contrast-enhanced CT findings (6th day after hospitalization): Multiple thrombosis from the descending aorta to the abdominal aorta (○). No obvious evidence of intestinal ischemia was observed

**Fig. 2:**
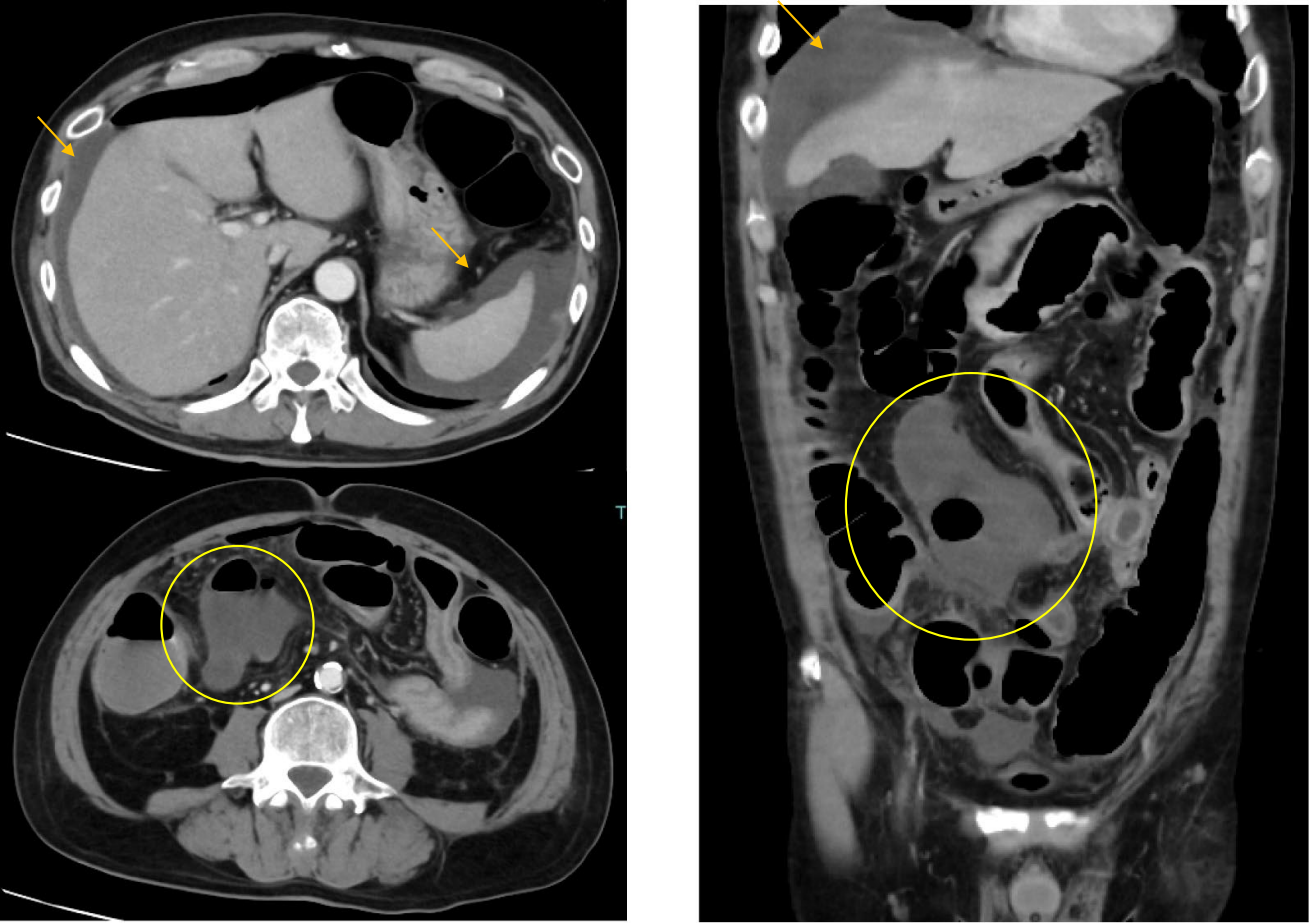
Abdominal contrast-enhanced CT findings. (10th day after hospitalization): Ileum ischemia(○) and ascites ( →)were observed

The patient was diagnosed with COVID-19 pneumonia and started on empiric therapy with tocilizumab (600 mg/day, Only administered on the first day.), remdesivir (200 mg/day on the first day, 100 mg/day on days 2 to 10) and dexamethasone (6.6 mg/day). His oxygen supplementation requirement increased gradually throughout the hospital stay, and Nasal High Flow™ was used to supply oxygen. Heparin (10,000 unit/day) was also administered for his high D-dimer level. He was taking orally and had no diarrhea or constipation. Diffuse abdominal distension and tenderness were noted on day 6 of his hospital stay, and his abdomen was evaluated. Contrast-enhanced CT revealed multiple thrombosis from the descending aorta to the abdominal aorta. No obvious evidence of intestinal ischemia was observed (Fig. 1). After CT showed descending aortic thrombus, heparin dose was increased to 12,000 unit/day. On day 10, the abdominal pain worsened, and a re-evaluation with contrast-enhanced CT led to the diagnosis of perforation peritonitis due to ischemic necrosis of the ileum (Fig. 2).

Vital signs were blood pressure 160/78 mmHg, heart rate 110 beats per minute, temperature 38.0 °C, SpO_2_ was 85% and PaO_2_ was 50 Torr (Nasal High Flow™ 30 L, 40%). His abdomen was distended and hard. He had oppressive pain at the hypogastrium and rebound tenderness.

### Blood test findings

The WBC count was high at 57,670/µl, but his CRP level was not high at 0.3 mg/dl. An arterial blood gas analysis did not reveal acidosis (Table 1). He was diagnosed with acute intestinal necrosis due to multiple thrombosis and underwent emergency surgery.

**Table 1:** Blood test findings and Physiology score (10th day after hospitalization)

WBC	57670/ul	CRP	0.3 mg/dl	A: Total Acute Physiology Score		Score
Neu	95.0%	CK	112 IU/l	Temperature	38 °C	0
Lym	2.0%	BUN	25 mg/dl	Mean arterial pressure (mmHg)	95	0
RBC	491×10^4^/ul	Cre	0.84 mg/dl	Heart rate (/min)	110	2
Hb	14.9g/dl	eGFR	71.2 ml/min	Respiratory rate (/min)	20	0
Hct	43.2%	Na	128 mEq/l	PaO_2_ (mmHg)	85.2	0
PLT	25.1×10^4^/ul	K	4.9 mEq/l	Arterial pH	7.409	0
		Cl	100 mEq/l	Serum HCO^3^ (mmol/l)	16.3	3
T-protein	6.6 g/dl			Serum Na (mEq/l)	128	2
Alb	2.5 g/dl	PT-INR	1.32	Serum K (mEq/l)	4.9	0
T bil	0.9 mg/dl	APTT	34 sec	Serum Cre (mEq/l)	0.84	0
D bil	0.3 mg/dL	FDP	9.7 μg/ml	Hct (%)	43.2	0
AST	25 IU/l	D-dimer	15.3 μg/mL	WBC (/ul)	57670	4
ALT	40 IU/l			Glasgow Coma Scale (GCS) Score＝15-GCS	0	0
ALP	276 IU/l	Lactic acid	33 mg/dl			
LDH	525 IU/l			B: Age	64	3
γ-GPT	58 IU/l			C: Chronic health status	none	0
AMY	444 IU/l			APACHEII Score (=A＋B＋C)	Total	14

### Surgical findings

**Fig. 3:**
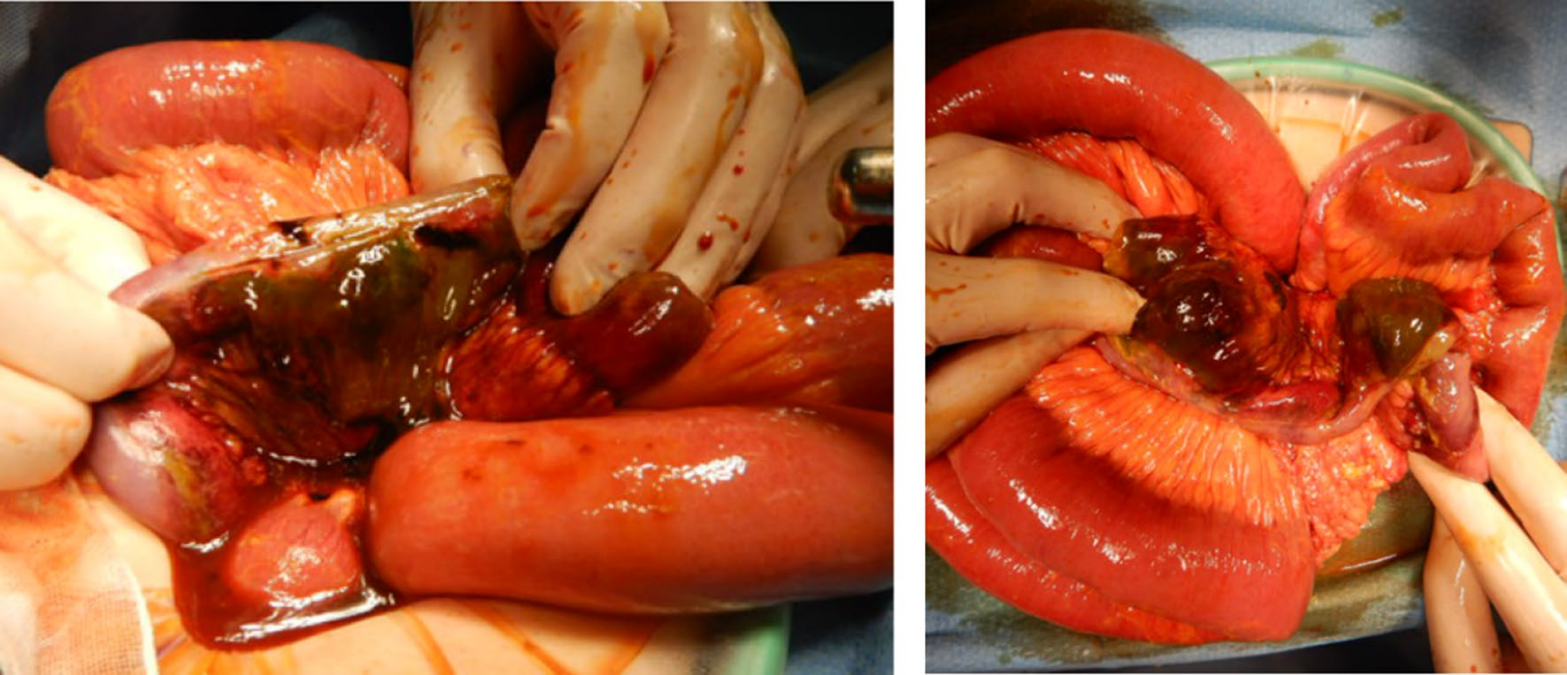
Intraoperative findings: Multiple necrotic perforations were found 25 to 40 cm before the ileocecal valve, so partial resection of the ileum (about 70 cm) was performed

**Fig. 4:**
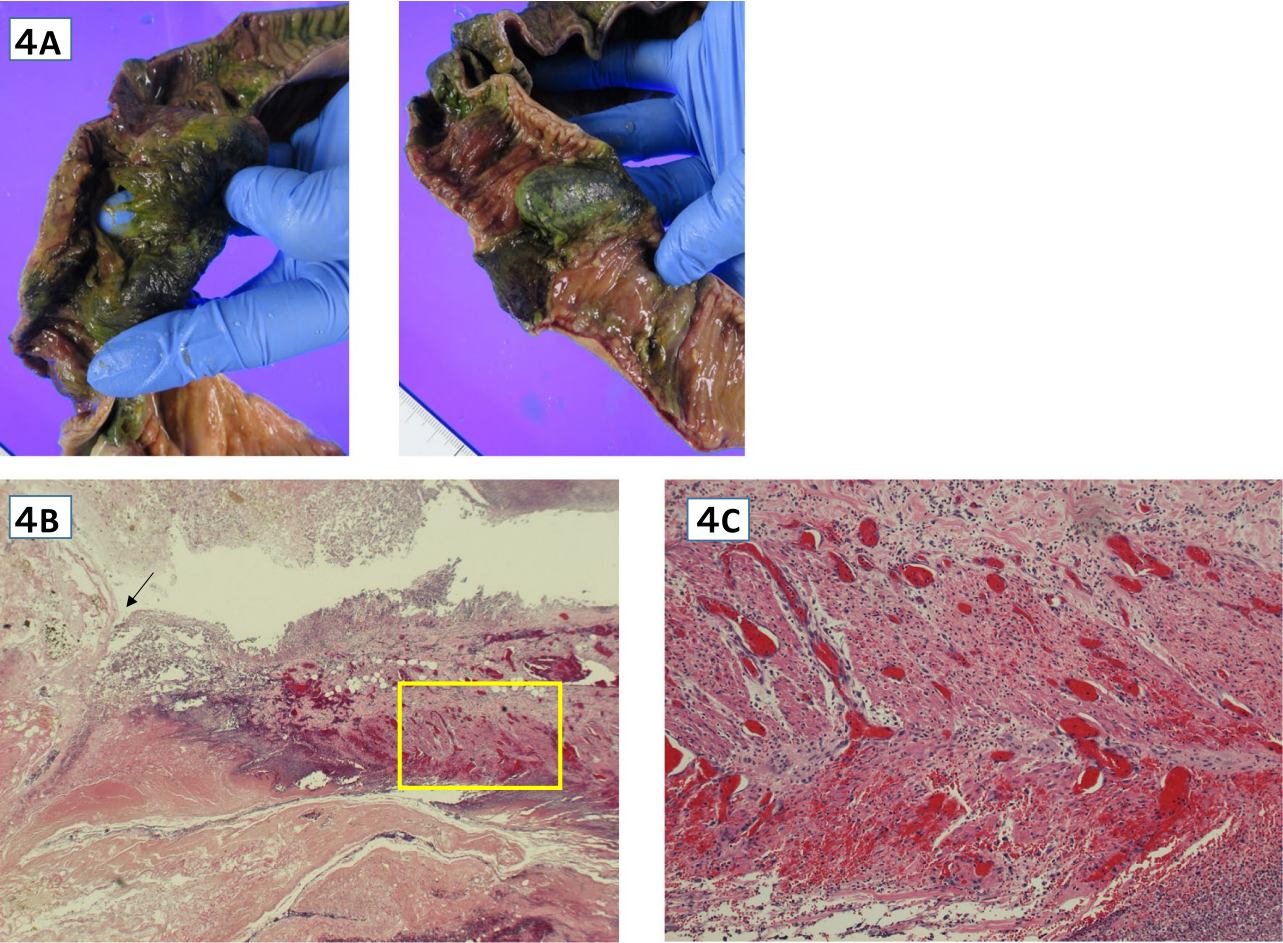
**A**–**C**: Pathological findings: **A** Multiple intestinal necrosis and perforation are observed in the ileum. **B**, **C** Frequent thrombi from the small intestinal mucosa around the perforation ( →) to the muscularis propria (HE staining × 20). **C** The muscular layer (□) in Fig. 4B is enlarged, thrombi that frequently occur in the muscularis peculiar to the small intestine around the perforation can be confirmed. (HE stain × 40)

Fecal contamination had spread throughout the abdominal cavity. Multiple necrotic perforations were found 25 to 40 cm before the ileocecal valve, so partial resection of the ileum (about 70 cm) was performed. Functional end-to-end anastomosis was performed using Endo GIA Universal linear staplers™. (Fig. 3). Pathological findings also revealed discontinuous multiple intestinal necrosis due to the frequent occurrence of thrombosis (Fig. 4A–C).

Postoperative course: His post-operative condition was poor, and he was kept ventilated in the intensive-care unit for six days. Antithrombotic therapy with Heparin (10,000 unit/day) and Edoxaban Tosilate Hydrate (30 mg/day)was started. Regarding pneumonia, a diffuse bilateral ground-glass like shadow and reticular shadow were observed upon chest CT at the time the patient was admitted. Although pleural effusions were observed post-surgery, they organized as time passed, and exhibited a trend towards improvement. His general condition gradually improved, and he was discharged 51 days after surgery. Contrast-enhanced CT before discharge revealed no further thrombus (Table 2).

**Table 2:**
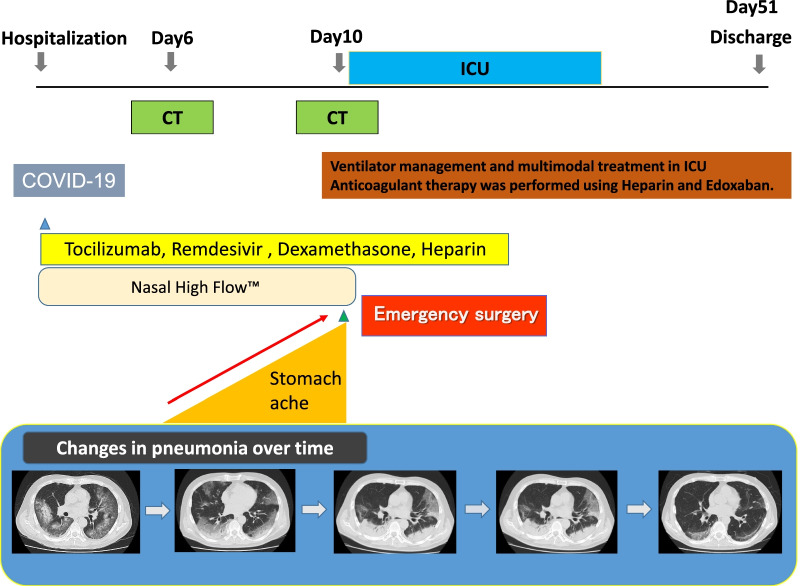
Progress after hospitalization

## Discussion

Since December, 2019, Wuhan, China, has experienced an outbreak of COVID-19, caused by the severe acute respiratory syndrome coronavirus 2 (SARS-CoV-2). Epidemiological and clinical characteristics of patients with COVID-19 have been reported but risk factors for mortality and a detailed clinical course of illness, including viral shedding, have not been well described [2].However, cases have been accumulated and the pathophysiology thereof has gradually been clarified. The most common cause of COVID-19 death is acute respiratory distress syndrome (ARDS) and the associated thrombosis of systemic organs. Acute mesenteric ischemia is a rare symptom for COVID-19 disease, so it is difficult to know the accurate occurrence thereof due to limitations in diagnostic imaging under strict disease management regulations. Studies have shown that thrombosis is associated with prognosis. The increase in D-dimer and FDP is a risk factor [3].The frequency of thrombosis by site was highest in the lower limb arteries (71%), followed by the upper limb arteries (14%), cerebral arteries (10%), and visceral arteries (4%) [4].In a report examining acute mesenteric ischemia of COVID-19 patients, small bowel ischemia (46.67%) was the most prevalent abdominal finding, followed by ischemic colitis (37.3%). Non-occlusive mesenteric ischemia (NOMI; 67.9%) indicating microvascular involvement was the most common pattern of bowel involvement. 50% of the patients receiving conservative/medical management died, highlighting high mortality without surgery [5].The mechanism of thrombosis is thought to be excessive complement system activity, inflammatory response, DIC status, vascular endothelitis, and cytokine storm [6].

There are two types of intestinal necrosis with COVID-19: those due to thrombosis; and those due to non-occlusive mesenteric ischemia. It has been reported that intestinal necrosis due to thrombosis occurs more frequently in patients who have not undergone anticoagulant therapy, with non-occlusive mesenteric ischemia occurring more frequently in severe cases. Non-occlusive mesenteric ischemia is a segmental disease that results in non-continuous intestinal blood flow obstruction in areas controlled by the main stem of the mesenteric vein, even with no organic obstructions [7]. In this case, because heparin was administered due to the patient being diagnosed with COVID-19 pneumonia, and because microvascular thrombosis was observed within the vein based on a pathological exam of the surgically removed small intestine, non-occlusive mesenteric ischemia was not the cause of the intestinal avascular necrosis. Although we were unable to confirm via CT that the blood clot causing the intestinal necrosis was from a clot in the aorta, as confirmed by CT, that had ruptured, it is possible that a microthrombus occurring in the ileum vessel caused the intestinal necrosis. If the clot in the aorta had ruptured, it is likely that many clot-derived events would have occurred in many organs, such as in the brain or lower limbs; however, this was not observed. Although we cannot completely rule out that the clot in the aorta had ruptured, we believe that the microthrombus which occurred in the ileum vessel led to a shower embolism, which in turn caused intestinal necrosis in multiple areas of the ileum, as intestinal necrosis was localized in the terminal ileal region.

The frequency of thrombosis was revealed by a Japanese domestic investigation. Among 6,202 COVID-19 inpatients, 108 cases (1.86%) of thromboembolism (24 cases of cerebral infarction, 7 cases of myocardial infarction, 41 cases of deep vein embolism, 30 cases of pulmonary thromboembolism, and 22 cases of others) were observed. In addition, the higher the severity, the higher the rate of thromboembolism [8]. Our hospital admitted a total of 1308 COVID-19 patients from February 8, 2020 to March 31, 2022. In addition to this case, we experienced 1 case of cerebral infarction, 2 cases of pulmonary thromboembolism, and 1 case of myocardial infarction. These all involved patients whose condition was classified as moderate or severe patients at the time of hospitalization. In this case, although the WBC count was always high due to the administration of steroids, CRP was not elevated due to the anti-inflammatory effect of tricizumab administration. Therefore, caution should be exercised regarding complications of serious infections in these patients. Regarding the treatment of COVID-19 thrombosis, it is stated in the < Guide to Medical Examinations for Coronavirus Disease 2019 Ver. 6 [9] > , that although the prophylactic dose of heparin has not been established, it should be a low dose (10,000 units/day). In this case, despite administering 10,000 units/day of heparin from the time of hospitalization, thromboembolism occurred; thus, the preventive effect of heparin seems to be limited. The accumulation of further evidence, such as when to switch to DOAC and when to discontinue anticoagulant therapy, is awaited.

## Conclusion

Although there are very few reports of intestinal necrosis due to thrombosis caused by COVID-19 on PubMed, it is anticipated that complications due to various thromboses will increase going forward due to the COVID-19 pandemic. Thrombosis due to COVID-19 complications is rare in the intestinal tract, but also occur. Its initial symptoms might not be captured by CT images, therefore caution is required.

Complications from thrombosis can be fatal and require prophylaxis, early diagnosis, and therapeutic intervention. We would like to report on a case in order to help with the future diagnosis and treatment of such patients.
